# Systematic review adherence to methodological or reporting quality

**DOI:** 10.1186/s13643-017-0527-2

**Published:** 2017-07-19

**Authors:** Kusala Pussegoda, Lucy Turner, Chantelle Garritty, Alain Mayhew, Becky Skidmore, Adrienne Stevens, Isabelle Boutron, Rafael Sarkis-Onofre, Lise M. Bjerre, Asbjørn Hróbjartsson, Douglas G. Altman, David Moher

**Affiliations:** 1 Ottawa Methods Centre, Clinical Epidemiology Program, Ottawa Hospital Research Institute, Centre for Practice-Changing Research, Ottawa, ON Canada; 2 Translational Research in Biomedicine (TRIBE) Program, University of Split School of Medicine, Split, Croatia; 3 Bruyère Research Institute, Ottawa, ON Canada; 4 Paris Descartes University, Centre of Research in Epidemiology and Statistics Sorbonne Paris Cité (CRESS), UMR 1153, INSERM, Paris, France; 5 Graduate Program in Dentistry, IMED, Passo Fundo, RS Brazil; 6 Department of Family Medicine, University of Ottawa, Ottawa, ON Canada; 7 School of Epidemiology, Public Health and Preventive Medicine, University of Ottawa, Ottawa, ON Canada; 8 Center for Evidence-Based Medicine, University of Southern Denmark/Odense University Hospital, Odense, Denmark; 9 Centre for Statistics in Medicine, Nuffield Department of Orthopaedics, Rheumatology and Musculoskeletal Sciences, University of Oxford, Oxford, UK; 10 Centre for Journalology; Canadian EQUATOR Centre, Clinical Epidemiology Program, Ottawa Hospital Research Institute, Centre for Practice-Changing Research, Ottawa, ON Canada

**Keywords:** Reporting quality, Methodological quality, Systematic reviews, Guideline adherence

## Abstract

**Background:**

Guidelines for assessing methodological and reporting quality of systematic reviews (SRs) were developed to contribute to implementing evidence-based health care and the reduction of research waste. As SRs assessing a cohort of SRs is becoming more prevalent in the literature and with the increased uptake of SR evidence for decision-making, methodological quality and standard of reporting of SRs is of interest. The objective of this study is to evaluate SR adherence to the Quality of Reporting of Meta-analyses (QUOROM) and PRISMA reporting guidelines and the A Measurement Tool to Assess Systematic Reviews (AMSTAR) and Overview Quality Assessment Questionnaire (OQAQ) quality assessment tools as evaluated in methodological overviews.

**Methods:**

The Cochrane Library, MEDLINE®, and EMBASE® databases were searched from January 1990 to October 2014. Title and abstract screening and full-text screening were conducted independently by two reviewers. Reports assessing the quality or reporting of a cohort of SRs of interventions using PRISMA, QUOROM, OQAQ, or AMSTAR were included. All results are reported as frequencies and percentages of reports and SRs respectively.

**Results:**

Of the 20,765 independent records retrieved from electronic searching, 1189 reports were reviewed for eligibility at full text, of which 56 reports (5371 SRs in total) evaluating the PRISMA, QUOROM, AMSTAR, and/or OQAQ tools were included. Notable items include the following: of the SRs using PRISMA, over 85% (1532/1741) provided a rationale for the review and less than 6% (102/1741) provided protocol information. For reports using QUOROM, only 9% (40/449) of SRs provided a trial flow diagram. However, 90% (402/449) described the explicit clinical problem and review rationale in the introduction section. Of reports using AMSTAR, 30% (534/1794) used duplicate study selection and data extraction. Conversely, 80% (1439/1794) of SRs provided study characteristics of included studies. In terms of OQAQ, 37% (499/1367) of the SRs assessed risk of bias (validity) in the included studies, while 80% (1112/1387) reported the criteria for study selection.

**Conclusions:**

Although reporting guidelines and quality assessment tools exist, reporting and methodological quality of SRs are inconsistent. Mechanisms to improve adherence to established reporting guidelines and methodological assessment tools are needed to improve the quality of SRs.

**Electronic supplementary material:**

The online version of this article (doi:10.1186/s13643-017-0527-2) contains supplementary material, which is available to authorized users.

## Background

Systematic reviews (SRs) are considered the gold standard for evidence used to evaluate the benefits and harms of healthcare interventions. They are powerful tools used to assess treatment effectiveness which can subsequently improve patient care [1]. SR evidence has become increasingly important in clinical decision-making and for informing clinical guidelines and health policy [2, 3].

Often, the quality of both methodology and reporting of SRs is flawed due to deficiencies in the design, conduct, and reporting. Poorly conducted SRs can lead to inaccurate estimates of treatment effectiveness, misleading conclusions, and reduced applicability, all of which are a waste of limited resources [4]. Unfortunately, poorly conducted or reported SRs may be associated with bias, limiting their usefulness [5]. When SRs comply with established methodology, report findings transparently, and are free of bias, they provide relevant information for practice guideline developers and other stakeholders such as policy makers [5]. As such, SR methodologists have proposed and developed various methodological and reporting guidelines over the years to assist in improving the methodological rigor and reporting of SRs.

With the rise of evidence-based medicine, criteria for assessing quality began to emerge, such as Mulrow [6] and Sacks [7]. In 1991, Oxman and Guyatt developed the Overview Quality Assessment Questionnaire (OQAQ) [8], a validated tool to assess methodological quality for SRs of intervention studies. Since then, SR methodologists have suggested several other methodological quality (MQ) items, such as potential sources of bias, as important in improving quality of conduct. A Measurement Tool to Assess Systematic Reviews (AMSTAR) [9] tool was developed in 2007 for SRs for intervention studies to include these additional items. In 2010, a revised tool (R-AMSTAR) was developed to provide a quantitative scoring method to assess quality [10]. The accurate reporting of methods and SR findings was established in the late 1990s. In 1999, the Quality of Reporting of Meta-analyses (QUOROM) Statement was developed to evaluate the completeness of reporting of meta-analyses of randomized trials [11]. A decade later, the Preferred Reporting Items for Systematic Reviews and Meta-Analyses (PRISMA) Statement was developed as an update of QUOROM to address several conceptual and methodological advances in the conduct and reporting of SRs of randomized trial [12]. In 2011, Cochrane developed the Methodological Expectations of Cochrane Intervention Reviews (MECIR) guidelines to specify the methodological and reporting standards for Cochrane intervention protocols and reviews [13, 14]. These guidelines drew criteria from AMSTAR, PRISMA, and other guidelines from organizations such as the US Institute of Medicine [13, 14].

Little was known about how quality or reporting of SRs was assessed in methodological reports. In a separate manuscript, we mapped the methods used to assess SR quality (e.g., use of quality assessment tools) or reporting of SRs (e.g., reporting guidelines) in methodological reports [15]. We found that the criteria used to assess MQ and reporting quality (RQ) of SRs varied considerably. These findings raised an important issue regarding how well SR authors used published reporting guidelines and MQ assessment tools.

Although methodological studies of SRs assessing the MQ or RQ have been published, adherence of SRs to established MQ and RQ assessment tools is unknown. We will address this aspect by examining existing methodological overviews.

### Objectives

The objective of this study was to determine SR adherence to the QUOROM and PRISMA reporting guidelines and the AMSTAR and OQAQ quality assessment tools as evaluated in methodological overviews.

## Methods

### Definitions and important concepts

SRs and meta-analyses were defined based on the guidelines provided by the Cochrane Collaboration and the PRISMA Statement [12, 16]. We adopted the term *overview* to mean a summary of evidence from more than one SR at a variety of different levels, including the combination of different interventions, different outcomes, different conditions, problems or populations, or the provision of a summary of evidence on the adverse events of an intervention [17, 18]. Other terminology used to describe overviews includes *systematic review of systematic reviews*, *reviews of reviews*, or an *umbrella review*. We included publications that are “methodological overviews,” meaning research that has assessed the MQ or RQ of a cohort of SRs and refer to these publications simply as “reports.”

#### Methodological quality and completeness of reporting

There is an important distinction between SR quality of methods and quality of reporting. MQ is concerned with how well a SR was designed and conducted (e.g., literature search, selection criteria, pooling of data). RQ refers to how well methodology and findings were described in the SR report(s) [19]. This critical difference should be reflected in the choice of quality assessment tools and reporting guidelines.

### Eligibility criteria

#### Inclusion criteria

This work stems from a parallel investigation where any methodological report published between January 1990 and October 2014 with a primary objective to assess the quality of methodology, reporting, or other quality characteristics of SRs was included [15]. We included only those methodological reports that evaluated SRs addressing the comparative effectiveness of interventions as most quality tools have been developed for intervention reviews. For this paper, however, we include only those reports using the most frequently employed published MQ (AMSTAR and OQAQ) and RQ (PRISMA and QUOROM) tools, as determined from the parallel investigation [15].

#### Exclusion criteria

We excluded reports of clinical interventions, where the intent was to summarize the evidence for use in healthcare decision-making; reports assessing the quality of diagnostic, screening, etiological, or prognostic studies; and other publication types, such as editorials, narrative reviews, rapid reviews, and network meta-analyses. Reviews that include study designs other than randomized controlled trials were also excluded. Reports in languages other than English were not included. Reports including fewer than 10 SRs, assessing the reliability of an assessment tool, evaluating only one methodological characteristic (e.g., search strategy), or those assessing only SRs with pooled estimates of effect were also excluded.

### Search methods

An experienced information specialist developed and conducted an extensive search of the Cochrane Library, EMBASE®, and MEDLINE® to identify methodological reports published between January 1990 and October 16, 2014. Potentially eligible titles and/or abstracts were identified using a combination of subject headings (e.g., “Meta-Analysis as Topic,” “Quality Control,” “Checklist”) and key words (e.g., “umbrella review,” scoring, compliance) (see Additional File 1). The search strategy was peer-reviewed prior to execution [20]. Additional reports eligible for inclusion were identified by members of the research team prior to the start of the project [2, 21, 22]. These articles were used as “seed” articles when developing the electronic search strategy.

### Screening

Titles and abstracts were screened for potentially relevant articles using a liberal accelerated approach (i.e., any potentially relevant citations were identified by one reviewer; a second person verified potential excludes). Full-text screening was completed independently and in duplicate by a team of reviewers with experience in methodological reviews; a 5% pilot testing was conducted at both screening levels. All screening disagreements were discussed among pairs of reviewers, with any outstanding disagreements resolved by an independent third reviewer (DM). A data management software, DistillerSR® [23], was used to manage retrieved records, screen citations/reports, record reasons for exclusion, and store extracted data.

### Data extraction

We developed standardized forms for data extraction of items of interest from the included reports. Basic characteristics and findings relating to the SRs that were reviewed were extracted from each included report by two of four reviewers; a 10% random sample of reports was assessed for accuracy. A pre-extraction meeting was held for all extraction levels along with pilot testing to ensure consistency across reviewers. The following basic characteristics of the included overviews were extracted: year of publication, number of included SRs, specified medical area, number of databases searched, language restrictions, SR definition, types of publishing journals, Cochrane or non-Cochrane review, reporting of availability of study protocol, and source of funding. Additional items pertaining to the evaluated reviews were extracted: intent of assessment (whether MQ or RQ), the method(s) used to assess MQ or RQ, and details of adherence of SRs to individual items included in OQAQ, AMSTAR, QUOROM, or PRISMA guidelines.

### Analyses

Summary statistics are reported as frequency and percentage of reports for report characteristics or frequency and percentage of compliant SRs. No formal inferential statistical analyses were conducted. In some cases, reports would allocate points, or scores, to MQ or RQ items. In these cases, we considered full points or a complete score to be optimal; any meeting partial scores would be considered non-adherent. A post hoc decision was made to look at publications by their intent to assess MQ only, RQ only, or both MQ and RQ. This decision was made without prior examination of the data by the senior investigator (DM). Due to the limited number of Cochrane reviews, the data did not allow for comparison of reports, including Cochrane versus non-Cochrane reviews, as planned. This study was not registered in PROSPERO or elsewhere as no known repositories take methodological protocols. However, the study protocol is available upon request.

## Results

**Fig. 1:**
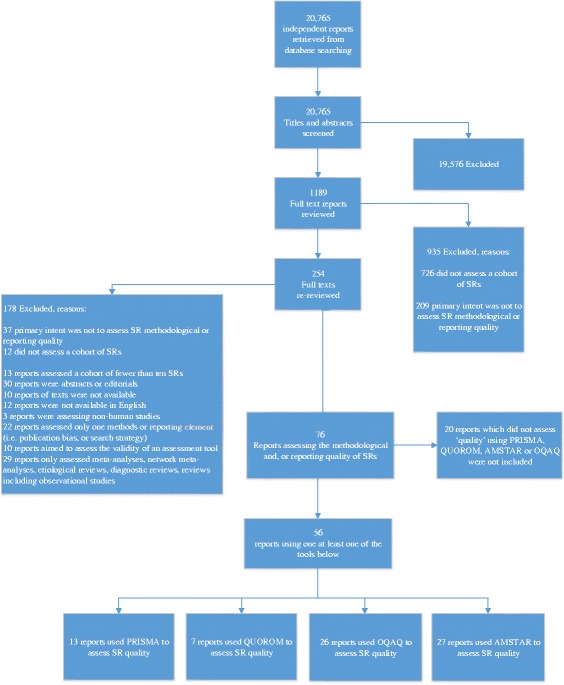
Flow of study reports

Of the 20,765 independent records retrieved from electronic searching, 1189 reports were reviewed in relation to a subset of the eligibility at full text, of which 935 were excluded for either not assessing a cohort of SRs or the primary intent was not to assess MQ or RQ. A secondary full-text review of the remaining 254 reports was carried out to determine whether exclusion criteria were met; 178 reports were excluded, leaving 76 potentially eligible reports. Once it was determined by the parallel investigation [15] which quality tools were used most often (OQAQ, AMSTAR, QUOROM, or PRISMA), 20 of the 76 reports were excluded for not using one of those tools. The tools or criteria used by the 20 reports were reported in a separate manuscript [15]. A total of 56 reports [21–77] evaluating 5371 SRs were included (Fig. 1).

### Report characteristics

The report characteristics are listed in Table 1. The majority of reports were conducted with the intent to assess MQ or RQ using an appropriate tool; 61% (34/56) of reports had a primary intent to assess MQ only, 7% (4/56) reported having a primary intent to assess RQ, and 27% (15/56) had a primary intent to assess both MQ and RQ. The remaining reports did not use the tools according to their intended use: one report used OQAQ for RQ assessment, one used PRISMA for both RQ and MQ assessments, and two reports used MQ tools to assess both MQ and RQ. Regardless of intent, 27 reports used AMSTAR, 26 reports used OQAQ, 13 reports used PRISMA, and seven reports used QUOROM.

**Table 1:** Table of characteristics by mechanism for assessing “quality”

Characteristic		Reports using PRISMA *N* = 13		Reports using QUOROM *N* = 7		Reports using OQAQ *N* = 26		Reports using AMSTAR *N* = 27		All reports *N* = 56	
		*n*	%	*n*	%	*n*	%	*n*	%	*n*	%
Year of publication of methodological report	1996–2010	0	0	7	100	20	77	0	0	21	38
	2010–2014	13	100	0	0	6	23	27	100	35	63
Number of assessed SRs across reports	Median (IQR)	88 (37, 134)		61 (53, 107)		59 (31, 109)		46 (22, 106)		57 (30, 109)	
	Range	10–487		10–161		10–200		10–369		10–487	
Were SRs of particular medical field?	No	1	8	2	29	2	8	1	4	5	9
	Yes	12	92	5	71	24	92	26	96	51	91
Intent of assessment	MQ tool for MQ assessment	–	–	–	–	16	62	18	67	34	61
	RQ tool for RQ assessment	2	15	2^a^	29	–	–	–	–	4	7
	Both MQ and RQ (and appropriate use of tool, accordingly)	10	77	5	71	7	27	8	30	15	27
	Used MQ tool for RQ assessment	–	–	–	–	1^a^	4	–	–	1	2
	Used MQ tool for both MQ and RQ assessment	–	–	–	–	1	4	–	–	1	2
	Used MQ tools plus other criteria; both MQ and RQ assessed^b^	–	–	–	–	1^c^	4	1^c^	4	1	2
	Used RQ tool for both MQ and RQ assessment	1	8	–	–	–	–	–	–	1	2
Cohort of Cochrane SRs	Cochrane only	0	0	3	43	4	15	0	0	4	7
	Sample of reviews	6	46	3	43	11	42	13	48	28	50
	Specific journal sample or other	7	54	1	14	11	42	14	52	24	43
Number of databases searched	1	2	15	4	57	4	15	4	15	10	18
	2	0	0	1	14	2	8	3	11	5	9
	3	1	8	1	14	6	23	1	4	7	13
	4	4	31	0	0	5	19	4	15	10	18
	5	1	8	1	14	5	19	4	15	8	14
	6	1	8	0	0	1	4	2	7	3	5
	7	1	8	0	0	1	4	2	7	3	5
	8+	0	0	0	0	2	8	1	15	3	5
	Not reported	0	0	0	0	0	0	2	7	2	4
	Not applicable (select journals)	3	23	0	0	0	0	4	15	5	9
Reports restricted SRs by language	No restrictions	2	15	2	29	12	46	4	15	15	27
	Not reported	7	54	4	57	10	39	13	48	22	39
	Restricted to English	1	8	1	14	6	23	7	26	13	23
	Restricted to English and other specified languages	3	23	0	0	0	0	3	11	6	11
SR defined for inclusion criteria	Not reported	2	15	1	14	7	27	6	22	12	21
	Yes, but no reference given	4	31	1	14	5	19	5	19	10	18
	“Systematic review” reported as a search term	5	39	4	57	13	50	9	33	24	43
	Cochrane Collaboration and PRISMA Statement	2	15	1	14	2	8	5	19	7	13
	Other reference	0	0	0	0	1	4	2	7	3	5
Was a study protocol reported as available for this report?	No or not reported	11	85	6	86	24	92	24	89	49	88
	Yes, link reported	2	15	0	0	1	4	1	4	2	4
	Yes, upon request	0	0	1	14	3	12	2	7	5	9
Report source of funding	Industry Funded	0	0	0	0	2	8	1	4	1	2
	Non-profit Funding	7	54	3	43	13	50	10	37	26	46
	Reported no funding	1	8	1	14	5	19	6	22	8	14
	Not reported	5	39	3	43	10	39	10	37	21	38

Note: columns are not mutually exclusive

^a^One study evaluated both QUOROM and OQAQ for RQ

^b^Unclear from the study description whether MQ tools and/or additional criteria were used to assess the RQ aspect of the study

^c^Same report

Reports spanned an 18-year period, of which 63% (35/56) were published between 2010 and 2014, indicating a marked increase in recent years. A median of 57 SRs (interquartile range 30 to 109) were assessed in reports. Almost all reports (91%) addressed SRs of a topic within a specific medical field. Forty-three percent (24/56) of reports include SRs limited to specific journals, half (28/56) included SRs from a general sample of reviews across medical journals, and only 7% (4/56) evaluated a cohort of Cochrane reviews (i.e., from one specific source). Accordingly, the majority of reports provided details for the source of SRs, whether it was databases or specific journals. Information as to whether language restrictions were used was provided in 61% (34/56) of reports. In relation to specifying a definition for SR, 21% (12/56) did not report this information. The majority of reports (88%) did not state whether a protocol was available. Thirty-eight percent (21/56) of reports did not state the source of funding for their research. Table 1 also details these characteristics according to reports using a particular tool.

### Adherence to MQ and RQ items in methodological reports

The reports assessed adherence to items for the most frequently used MQ and RQ tools (i.e., AMSTAR, OQAQ, QUOROM, PRISMA). These data have been collated across the samples of SRs (Tables 2, 3, 4, and 5). Data pertaining to adherence to quality or reporting criteria by item were obtainable from most methodological reports: 100% (13/13) using PRISMA, 71% or more (5–6 out of 7, depending on the item) using QUOROM, 85% or more (22–23 out of 27, depending on the item) using AMSTAR, and 85% (22/26) using OQAQ.

**Table 2:** Summary across reports of systematic reviews adhering to PRISMA reporting guidelines (*N* = 13)

Item assessed	Item description	No. of reports reporting adherence by item	Adhering SRs	Total SRs	%
1. Title	Identify the report as a systematic review, meta-analysis, or both	13	1480	1741	85
2. Abstract: structured summary	Provide a structured summary including the following as applicable: background; objectives; data sources; study eligibility criteria, participants, and interventions; study appraisal and synthesis methods; results; limitations; conclusions and implications of key findings; systematic review registration number	13	885	1741	51
3. Introduction: rationale	Describe the rationale for the review in the context of what is already known	13	1532	1741	88
4. Objectives	Provide an explicit statement of questions being addressed with reference to participants, interventions, comparisons, outcomes, and study design (PICOS)	13	1039	1741	60
5. Methods: protocol and registration	Indicate if a review protocol exists, if and where it can be accessed (e.g., web address), and, if available, provide registration information including registration number	13	102	1741	6
6. Eligibility criteria	Specify study characteristics (e.g., PICOS, length of follow-up) and report characteristics (e.g., years considered, language, publication status) used as criteria for eligibility, giving rationale	13	1342	1741	77
7. Information sources	Describe all information sources (e.g., databases with dates of coverage, contact with study authors to identify additional studies) in the search and date last searched	13	1530	1741	88
8. Search	Present full electronic search strategy for at least one database, including any limits used, such that it could be repeated	13	923	1741	53
9. Study selection	State the process for selecting studies (i.e., screening, eligibility, included in systematic review, and, if applicable, included in the meta-analysis)	13	1048	1741	60
10. Data collection process	Describe method of data extraction from reports (e.g., piloted forms, independently, in duplicate) and any processes for obtaining and confirming data from investigators	13	1059	1741	61
11. Data items	List and define all variables for which data were sought (e.g., PICOS, funding sources) and any assumptions and simplifications made	13	865	1741	50
12. Risk of bias in individual studies	Describe methods used for assessing risk of bias of individual studies (including specification of whether this was done at the study or outcome level) and how this information is to be used in any data synthesis	13	1251	1741	72
13. Summary measures	State the principal summary measures (e.g., risk ratio, difference in means)	13	1353	1741	78
14. Synthesis of results	Describe the methods of handling data and combining results of studies, if done, including measures of consistency (e.g., *I* ^2^) for each meta-analysis	13	1129	1736	65
15. Risk of bias across studies	Specify any assessment of risk of bias that may affect the cumulative evidence (e.g., publication bias, selective reporting within studies)	13	657	1741	38
16. Additional analyses	Describe methods of additional analyses (e.g., sensitivity or subgroup analyses, meta-regression), if done, indicating which were pre-specified	13	879	1738	51
17. Results: study selection	Give numbers of studies screened, assessed for eligibility, and included in the review, with reasons for exclusions at each stage, ideally with a flow diagram	13	1094	1740	63
18. Study characteristics	For each study, present characteristics for which data were extracted (e.g., study size, PICOS, follow-up period) and provide the citations	13	1324	1741	76
19. Risk of bias within studies	Present data on risk of bias of each study and, if available, any outcome level assessment (see item 12)	13	1199	1738	69
20. Results of individual studies	For all outcomes considered (benefits or harms) present for each study: (a) simple summary data for each intervention group and (b) effect estimates and confidence intervals, ideally with a forest plot	13	1399	1737	81
21. Synthesis of results	Present results of each meta-analysis done, including confidence intervals and measures of consistency	13	1150	1687	68
22. Risk of bias across studies	Present results of any assessment of risk of bias across studies (see item 15)	13	527	1736	30
23. Additional analysis	Give results of additional analyses, if done (e.g., sensitivity or subgroup analyses, meta-regression [see item 16])	13	631	1658	38
24. Discussion: summary of evidence	Summarize the main findings including the strength of evidence for each main outcome; consider their relevance to key groups (e.g., healthcare providers, users, and policy makers)	13	1085	1741	62
25. Limitations	Discuss limitations at study and outcome level (e.g., risk of bias) and at review-level (e.g., incomplete retrieval of identified research, reporting bias)	13	1358	1741	78
26. Conclusions	Provide a general interpretation of the results in the context of other evidence and implications for future research	13	1480	1741	85
27. Funding	Describe sources of funding for the systematic review and other support (e.g., supply of data) and role of funders for the systematic review	13	647	1741	37

**Table 3:** Summary across reports of systematic reviews adhering to QUOROM reporting guideline (*N* = 7)

Item assessed	Item description	No. of reports reporting adherence by item	Adhering SRs	Total SRs	%
Title	Identify the report as a systematic review	6	133	449	30
Abstract	Use a structured format	6	402	449	90
	Describe the clinical question explicitly	6	341	449	76
	Describe the databases (i.e., list) and other information sources	6	335	449	75
	Describe the selection criteria (i.e., population, intervention, outcome, and study design), methods for validity assessment, data abstraction, and study characteristics, and quantitative data synthesis in sufficient detail to permit replication	5	177	388	46
	Describe characteristics of the RCTs included and excluded; qualitative and quantitative findings (i.e., point estimates and confidence intervals); and subgroup analyses	5	180	388	46
	Describe the main results	6	425	449	95
Introduction: rationale	Describe the explicit clinical problem, biological rationale for the intervention, and rationale for review	6	382	449	85
Search	Describe the information sources, in detail (e.g., databases, registers, personal files, expert informants, agencies, hand-searching), and any restrictions (years considered, publication status, language of publication)	5	274	388	71
Study selection	Describe the inclusion and exclusion criteria (defining population, intervention, principal outcomes, and study design)	6	417	449	93
Data collection process	Data extraction: describe the process or processes used (e.g., completed independently, in duplicate)	6	363	449	81
Data items	Describe the type of study design, participants’ characteristics, details of intervention, outcome definitions, and how clinical heterogeneity was assessed	6	316	449	70
Risk of bias in individual studies	Validity assessment: describe the criteria and process used (e.g., masked conditions, quality assessment, and their findings)	6	240	449	54
Synthesis of results	Describe the principal measures of effect (e.g., relative risk), method of combining results (statistical testing and confidence intervals), handling of missing data; how statistical heterogeneity was assessed; a rationale for any a priori sensitivity and subgroup analyses; and any assessment of publication bias	5	219	388	56
Results: study selection	Provide a meta-analysis profile summarizing trial flow	6	40	449	9
Study characteristics	Present descriptive data for each trial (e.g., age, sample size, intervention, dose, duration, follow-up period)	6	384	449	86
Results of individual studies	Report agreement on the selection and validity assessment; present simple summary results (for each treatment group in each trial, for each primary outcome); present data needed to calculate effect sizes and confidence intervals in intention-to-treat analyses (e.g., 2 × 2 tables of counts, means and SDs, proportions)	5	213	388	55
Discussion: summary of evidence	Summarize key findings; discuss clinical inferences based on internal and external validity; interpret the results in light of the totality of available evidence; describe potential biases in the review process (e.g., publication bias); and suggest a future research agenda	5	265	388	68

**Table 4:** Summary across reports of systematic reviews meeting AMSTAR quality assessment criteria (*N* = 27)

Item assessed	Item Description	No. of reports reporting adherence by item	Adhering SRs	Total SRs	%
1. Methods: Protocol and registration	Was an 'a priori' design provided?	23	820	1794	46
2. Information sources	Was the status of publication (i.e. grey literature) used as an inclusion criterion?	23	1013	1794	57
3. Search	Was a comprehensive literature search performed?	23	1149	1794	64
4. Data collection process	Was there duplicate study selection and data extraction?	23	534	1794	30
5. Results: Study selection	Was a list of studies (included and excluded) provided?	22	537	1779	30
6. Study characteristics	Were the characteristics of the included studies provided?	23	1439	1794	80
7. Risk of bias within studies	Was the scientific quality of the included studies assessed and documented?	23	1200	1794	67
8. Synthesis of results	Were the methods used to combine the findings of studies appropriate?	23	1169	1794	65
9. Risk of bias across studies	Was the likelihood of publication bias assessed?	23	995	1794	56
10. Limitations	Was the scientific quality of the included studies used appropriately in formulating conclusions?	23	590	1794	33
11. Funding	Was the conflict of interest stated?	22	685	1779	39

**Table 5:** Summary across reports of systematic reviews adhering to OQAQ items (*N* = 26)

Item assessed	Item description	No. of reports reporting adherence by item	Adhering SRs	Total SRs	%
1. Information sources	Were the search methods used to find evidence reported?	22	1027	1387	74
2. Search	Was the search strategy for evidence reasonably comprehensive?	22	754	1370	55
3. Study selection	Were the criteria used for deciding which studies to include in the overview reported?	22	1112	1387	80
4. Risk of bias in individual studies	Were criteria used for assessing validity of the included studies reported?	22	499	1367	37
5. Synthesis of results	Were findings of the relevant studies combined appropriately relative to the primary question addressed?	22	830	1387	60
6. Results: study selection	Was bias in the selection of studies avoided?	22	740	1351	55
7. Synthesis of results	Were methods used to combine the findings of relevant studies (to reach a conclusion) reported?	22	1005	1387	73
8. Limitations	Was the validity of all studies referred to in the text assessed using appropriate criteria (either in selecting studies for inclusion or in analyzing studies that are cited)?	22	898	1363	66
9. Conclusions	Were the conclusions made by the author (s) supported by the data and/or analysis reported in the overview?	22	1076	1387	78

### Adherence to reporting guidelines (RQ)

A total of 1741 SRs were included in the 13 reports that used PRISMA (Table 2). Over 85% of SRs fully reported their title, provided a rationale for the review, described all information sources, and provided a general interpretation of the results. However, compliance was poor for several items, with only 38% (657/1741) of SRs specifying any assessment of risk of bias methods across studies, 30% (527/1736) presenting results of risk of bias assessments across studies, and 37% (647/1741) describing sources of funding. Less than 6% (102/1741) provide protocol information in their SR report.

Six reports evaluating 449 SRs used QUOROM (Table 3). One additional report did not provide any information by item and is excluded from the analysis. Thirty percent (133/449) identified the report as a systematic review, and 9% (40/449) of SRs provided a figure summarizing trial flow. Included SRs adhered well to several QUOROM items. Over 85% of SRs used a structured format in the abstract, described the main results in the abstract, provided an explicit clinical question and rationale in the introduction/background section, described the study selection criteria, and presented descriptive data for each trial.

### Adherence according to methodological quality

A total of 1794 SRs were included in the 23 reports that provided AMSTAR assessments by item (Table 4). Eighty percent (1439/1794) of SRs provided the characteristics of included studies. Just over half (995/1794) assessed publication bias. Thirty-nine percent (685/1779) stated a conflict of interest, and a third (590/1794) of SRs reported limitations. In addition, 30% (534/1794) of SRs used duplicate study selection and data extraction during the data collection process and 30% (537/1779) provided a list of included and excluded studies.

Twenty-two reports evaluating 1387 SRs used the OQAQ criteria (Table 5). Thirty-seven percent (499/1367) of the SRs assessed risk of bias (validity) in the included studies. Comparatively, 80% (1112/1387) of the SRs reported the criteria for study selection, 75% (1027/1387) of SRs reported search methods used to find the evidence, 73% (1005/1387) described the methods used to combine the findings, and 78% (1076/1387) of SRs determined whether the conclusions were supported by the data.

## Discussion

Previously, we identified that the most commonly used tools or guidelines for critical appraisal and RQ assessment were QUOROM, PRISMA, AMSTAR, and OQAQ [15]. In this study, we evaluated SR, MQ, or RQ adherence to these quality assessments or reporting guidelines tools across methodological reports published between 1990 and 2014.

Our results indicate that SR adherence to reporting items was variable. Over 85% provided a rationale for the review when assessed using PRISMA, yet less than 6% gave protocol information in their SR report. Our study, like others, shows that reporting of review protocols is poorly reported [2, 24]. Review protocols are important to reduce duplication of research, allow researchers to plan and anticipate potential issues, assess validity of methods and replication of the review if desired, and prevent arbitrary decision-making [78, 79]. In addition, risk of bias across individual studies within reviews, additional analyses, and funding source were also poorly reported. These findings are consistent with other research [24]. We note that compliance to some reporting criteria has improved over time. Nine percent provided a trial flow diagram as reported using the QUOROM guidelines, compared to 63% using the PRISMA guidelines. This observed improvement in reporting could be partly due to journal endorsement of the reporting guideline but also due to authors’ exposure to the published tools or their general awareness to the issues of reporting in health research over time. For the few items that are similar between PRISMA and QUOROM and show a lower compliance with PRISMA, these results are possibly attributed to differences in operationalization of the criteria or simply as chance findings.

Adherence to methodological quality items was also variable. Overall, SRs using OQAQ adhered quite well to all methodological items in the tool. OQAQ was validated and is well accepted, but it was developed and validated over two decades ago [8]. The OQAQ criteria do not include assessment of issues such as a priori design, assessment of publication bias, and conflict of interest. As such, OQAQ differs from AMSTAR, which was published and validated more recently [80, 81]. For the 27 reports using AMSTAR to assess quality of SRs, the percentage of SRs meeting AMSTAR criteria was mediocre. One third or less of SRs used duplicate study selection and data extraction, provided a list of included and excluded studies within their review, or reported limitations. One small study has also shown the need for better adherence to AMSTAR [82]. We would expect that future research will include an evaluation of the recently published risk of bias in systematic reviews (ROBIS) tool [83].

SR evidence is used by decision-makers, policy makers, and other stakeholders. They should expect consistent and high-quality standards for reporting and conduct. Guidelines and tools have been developed over the years to improve RQ and MQ of SRs. Our findings suggest that for several items in MQ or RQ tools, SR authors comply well with the guidelines, but some items require major improvement. Other studies have also found that methodological and reporting quality is suboptimal [2, 84, 85]. In addition, evidence is emerging that biases within SRs could influence results and quality of overviews [86]. Effort should be directed towards improving the quality and reporting of SRs, wherever possible.

Journal endorsement and implementation of the use of reporting guidelines and critical appraisal tools during the editorial process is one mechanism to facilitate better quality. There is insufficient evidence to date in relation to systematic reviews but some information in relation to trials. One recent methodological review found insufficient evidence to determine a relationship between endorsement and completeness of reporting: Of 101 reporting guidelines, only seven had evaluable data from only a few evaluations each [87]. One small study found that reporting and methodological quality (adherence to both AMSTAR and PRISMA) significantly increased after journal endorsement of the PRISMA guidelines [25]. Readers may also be curious as to whether reporting differs when examining the influence of publication of the tools, such as a before and after publication comparison; none of the included methodological reviews assessed this. Further, in thinking about publication and then journal endorsement as potential interventions, we would agree with previously published work that journal endorsement might serve as a “stronger” intervention [87].

One unexplored hypothesis is whether the endorsement and use of reporting tools at the protocol phase of a SR paves the way for better reporting and methodological quality for the SR report. Review protocols allow researchers to plan and anticipate potential issues, assess validity of methods, and prevent arbitrary decision-making [78, 79]. The reporting of protocols can be guided and assessed by the Preferred Reporting Items for Systematic Reviews and Meta-Analysis for Protocols 2015 (PRISMA-P 2015) [78, 79]. Further, Moher et al. [2] suggested that granting agencies and journals require full compliance with established reporting and methodological guidelines, such as a requirement to include SR protocols with the submission of a SR.

Our review was limited exclusively to SRs included by authors of methodological reports. Each overview had their own selection criteria and quality thresholds; therefore, we did not seek out the publication of the individual SRs but relied on the data reported in each overview. As such, there is inherent heterogeneity that may be causing some of the observed variation in MQ and RQ. In addition, we relied on how the authors assessed and reported adherence. Variability in how strictly review authors assessed adherence to items in MQ and RQ tools could result in additional heterogeneity. Nevertheless, this report provides some insight into the adherence to quality assessment and reporting guideline items.

A rigorous development of tools for MQ and RQ is important and should involve several steps and appropriate consideration of stakeholders and methodological experts’ participation [88]. Despite considerable effort, the delivery of fit-for-purpose tools may not always be optimally achieved if items are not completely reflective of intent. For example, it could be reasonable to note that some MQ items in both AMSTAR and OQAQ are written in language that reflects more of reporting than conduct. We encourage developers to carefully consider the wording of items. Further, any tool could potentially be subject to content modifications as the science of health research methodology continues to evolve.

## Conclusions

In conclusion, the methodological and reporting quality of SRs varied considerably across items in four well-known tools. Mechanisms to improve adherence to established reporting guidelines and methodological assessment tools are needed to improve the quality of SRs.

## Additional file

Additional file 1: Search strategy. (DOCX 16 kb)

## Abbreviations

AMSTAR: A Measurement Tool to Assess Systematic Reviews
MECIR: Methodological Expectations of Cochrane Intervention Reviews
MQ: Methodological quality
OQAQ: Overview Quality Assessment Questionnaire
PRISMA: Preferred Reporting Items for Systematic Reviews and Meta-Analyses
PRISMA-P: Preferred Reporting Items for Systematic Reviews and Meta-Analysis for Protocols
QUOROM: Quality of Reporting of Meta-analyses
R-AMSTAR: Revised-A Measurement Tool to Assess Systematic Reviews
RQ: Reporting quality
SR: Systematic review
