# Supplementary material for: Systematic review adherence to methodological or reporting quality
Source: Syst Rev. 2017 Jul 19;6:131. doi: 10.1186/s13643-017-0527-2 (PMC5516390; doi:10.1186/s13643-017-0527-2)
Supplement: Additional file 1: — Search strategy. (DOCX 16 kb) [file 13643_2017_527_MOESM1_ESM.docx]

**Additional File 1. Search Strategy**

**OVERVIEWS Project - Update**

2014 Oct 16 - Final

Database: Ovid MEDLINE(R) In-Process & Other Non-Indexed Citations and Ovid MEDLINE(R) <1946 to Present>, Embase <1980 to 2014 Week 41> Search Strategy:

--------------------------------------------------------------------------------

1 exp meta-analysis as topic/ (30117)

2 exp "Review Literature as Topic"/ (54774)

3 ((review$1 or overview$1) adj2 synthes$2).tw. (2346)

4 (umbrella review$1 or meta-review$1 or metareview$1).tw. (214)

5 ("review of reviews" or "reviews of reviews" or "overview of reviews" or "overviews of reviews" or "overview of overviews" or "overviews of overviews").tw. (941)

6 ("review of systematic reviews" or "reviews of systematic reviews" or "review of systematic overviews" or "reviews of systematic overviews" or "overview of systematic reviews" or "overviews of systematic reviews" or "systematic review of systematic reviews" or "systematic reviews of systematic reviews").tw. (737)

7 or/1-6 (87156)

8 exp Quality Control/ (307341)

9 exp Guideline Adherence/ (342453)

10 exp Checklist/ (11304)

11 st.fs. (570230)

12 (quality or standard* or criteri* or characteristic* or guideline* or guidance* or checklist* or check list* or score$1 or scoring).tw. (6785515)

13 (adher* or comply$ or compli$2 or complian* or conform* or evaluat* or assess*).tw. (8945666)

14 or/8-13 (13436747)

15 7 and 14 (26070)

16 limit 15 to yr="1990 -Current" (25694)

17 (2012* or 2013* or 2014*).ed. (2887191)

18 16 and 17 (2488)

19 18 use prmz (2488)

20 exp "meta analysis (topic)"/ (15619)

21 exp "systematic review (topic)"/ (8563)

22 ((review$1 or overview$1) adj2 synthes$2).tw. (2346)

23 (umbrella review$1 or meta-review$1 or metareview$1).tw. (214)

24 ("review of reviews" or "reviews of reviews" or "overview of reviews" or "overviews of reviews" or "overview of overviews" or "overviews of overviews").tw. (941)

25 ("review of systematic reviews" or "reviews of systematic reviews" or "review of systematic overviews" or "reviews of systematic overviews" or "overview of systematic reviews" or "overviews of systematic reviews" or "systematic review of systematic reviews" or "systematic reviews of systematic reviews").tw. (737)

26 or/20-25 (25131)

27 exp quality control/ (307341)

28 exp standard/ (368330)

29 exp checklist/ (11304)

30 (quality or standard* or criteri* or characteristic* or guideline* or guidance* or checklist* or check list* or score$1 or scoring).tw. (6785515)

31 (adher* or comply$ or compli$2 or complian* or conform* or evaluat* or assess*).tw. (8945666)

32 or/27-31 (13196105)

33 26 and 32 (13486)

34 limit 33 to yr="1990 -Current" (13442)

35 (2012* or 2013* or 2014*).em. (6901947)

36 34 and 35 (10044)

37 36 use emez (9691)

38 19 or 37 (12179)

39 limit 38 to yr="2014-current" (3055)

40 remove duplicates from 39 (2926)

41 limit 38 to yr="2013" (4595)

42 remove duplicates from 41 (4114)

43 limit 38 to yr="2012" (3459)

44 remove duplicates from 43 (3058)

45 40 or 42 or 44 (10098)

46 45 use prmz (1929)

47 45 use emez (8169)

***************************

Search Name: Quality of Systematic Reviews

Date Run: 16/10/14 15:41:42.930

Description: 2012 May 18

ID Search Hits

#1 MeSH descriptor: [Meta-Analysis as Topic] explode all trees 553

#2 MeSH descriptor: [Review Literature as Topic] explode all trees 135

#3 ((review* or overview*) near/2 synthes*):ti,ab,kw 112

#4 (umbrella review* or meta-review* or metareview*):ti,ab,kw 23

#5 ("review of reviews" or "reviews of reviews" or "overview of reviews" or "overviews of reviews" or "overview of overviews" or "overviews of overviews"):ti,ab,kw 42

#6 ("review of systematic reviews" or "reviews of systematic reviews" or "review of systematic overviews" or "reviews of systematic overviews" or "overview of systematic reviews" or "overviews of systematic reviews" or "systematic review of systematic reviews" or "systematic reviews of systematic reviews"):ti,ab,kw 41

#7 #1 or #2 or #3 or #4 or #5 or #6 868

#8 MeSH descriptor: [Quality Control] explode all trees 497

#9 MeSH descriptor: [Guideline Adherence] explode all trees 730

#10 MeSH descriptor: [Checklist] explode all trees 81

#11 Any MeSH descriptor with qualifier(s): [Standards - ST] 11461

#12 (quality or standard* or criteri* or characteristic* or guideline* or guidance* or checklist* or score$1 or scoring):ti,ab,kw 216378

#13 (adher* or comply* or compli* or complian* or conform* or evaluat* or assess*):ti,ab,kw 362963

#14 #8 or #9 or #10 or #11 or #12 or #13 437877

#15 #7 and #14 461

#16 #15 Publication Year from 2012 to 2014 83
